# Lead Discovery for Alzheimer's Disease Related Target Protein RbAp48 from Traditional Chinese Medicine

**DOI:** 10.1155/2014/764946

**Published:** 2014-06-02

**Authors:** Hung-Jin Huang, Cheng-Chun Lee, Calvin Yu-Chian Chen

**Affiliations:** ^1^Department of Chinese Pharmaceutical Sciences and Chinese Medicine Resources, College of Pharmacy, China Medical University, Taichung 40402, Taiwan; ^2^School of Medicine, College of Medicine, China Medical University, Taichung 40402, Taiwan; ^3^Department of Biomedical Informatics, Asia University, Taichung 41354, Taiwan

## Abstract

Deficiency or loss of function of Retinoblastoma-associated proteins (RbAp48) is related with Alzheimer's disease (AD), and AD disease is associated with age-related memory loss. During normal function, RbAp48 forms a complex with the peptide FOG-1 (friend of GATA-1) and has a role in gene transcription, but an unstable complex may affect the function of RbAp48. This study utilizes the world's largest traditional Chinese medicine (TCM) database and virtual screening to provide potential compounds for RbAp48 binding. A molecular dynamics (MD) simulation was employed to understand the variations after protein-ligand interaction. FOG1 was found to exhibit low stability after RbAp48 binding; the peptide displayed significant movement from the initial docking position, a phenomenon which matched the docking results. The protein structure of the other TCM candidates was not variable during MD simulation and had a greater stable affinity for RbAp48 binding than FOG1. Our results reveal that the protein structure does not affect ligand binding, and the top three TCM candidates Bittersweet alkaloid II, Eicosandioic acid, and Perivine might resolve the instability of the RbAp48-FOG1 complex and thus be used in AD therapy.

## 1. Introduction

Alzheimer's disease (AD) is the most common neurodegenerative disease to occur in people around the ages of 65 to 69 years [1], but it is not a normal part of aging and younger people may also suffer from AD disease [2–4], although these cases are not common. AD symptoms involve memory loss, cognitive impairment which affects the ability to study, a reduction in activities, feeling loss, and long-term memory loss. The major neuropathology hallmarks are deposition of neuritic plaques and neurofibrillary tangles in the AD brain [5, 6]. Genetic mutations are the known causes of AD disease [7], with mutations occurring inthe genes for the amyloid precursor protein (APP). Presenilin 1 and presenilin 2 (PS1 and PS2) enhance the processing of transmembrane APP cleaved by alpha and beta proteases and gamma-secretases to form beta-amyloid 42 [8], which subsequently results in the development of AD. In curing this disease, in animal models, antiamyloid therapies were used to clear amyloid accumulation. However, recent strategies have not been successful in human AD patients. Scientists still do not fully understand the causes of AD disease, because of the existence of more than one high risk factor for neuronal dysfunction. In recent studies, Pavlopoulos et al. have demonstrated that Retinoblastoma-associated protein (RbAp48) deficiency or loss of function in the dentate gyrus (DG) is related to age-related memory loss [9]. RbAp48, which is a member of the NuRD (nucleosome remodeling and deacetylase) complex, is a histone-binding protein that targets chromatin assembly factors. NuRD is associated with gene expression and the presence of histone deacetylases for regulating transcription repressors [10, 11]. The transcription activation and repression of NuRD is regulated by FOG-1 (friend of GATA-1) which binds to RbAp48.

Traditional Chinese medicine (TCM) has been developed in China over thousands of years, and includes herbal medicine, acupuncture, Cupping, and Qigong. Traditional Chinese medicine has been used for stroke prevention [12–14], and in the treatment of cancer [15]. In this research, computer-aided drug design (CADD) is utilized. CADD has been widely used in many drug design studies [16, 17] which include molecular modeling approaches [18] and web server calculation [19, 20]. TCM is widely used in clinical treatment because of low side effects and low toxicity [21–23], and some studies have used extracts of Chinese herbs to investigate the therapeutic value of potential drugs [24, 25]. Research of CADD and TCM has been performed in many studies, such as influenza therapy [26–28], stroke prevention [29, 30], treatment of erectile dysfunction [31], reducing weight [32, 33], type II diabetes therapy [34], diseases associated with aging treatment [35], inflammation inhibitors development [36], HIV treatment [22], Parkinson's disease prevention [37], and cancer therapy [12, 38, 39]. Hence, we present a small molecule from the world's largest TCM database [40] to bind to RbAp48 and provide a more potent compound for target protein (RbAp48) binding than FOG-1.

## 2. Materials and Methods

### 2.1. Protein Preparation

The crystal structure of RbAp48 was taken from the PDB database (PDB code: 3RIK) [41]; the missing atoms and protonation were cleaned up by* Prepare Protein module* under Accelrys Discovery Studio 2.5.5.9350 (DS 2.5) [42]. All residues were protonated at pH 7.4. For structure validation, PONDR-FIT [43] was carried out to predict unfolded regions on the RbAp48 sequence.

### 2.2. Docking Analysis

Database screening involved 61,000 TCM compounds from the TCM Database@Taiwan (http://tcm.cmu.edu.tw/) being used for docking analysis, with the drug-likeness of all compounds being assessed by ADMET prediction. The LigandFit module in DS 2.5 was utilized to generate different ligand poses by Monte-Carlo techniques. All the ligand conformations were minimized by the CHARMm force field. The Ligand-receptor interaction energies were calculated by the DREIDING force field during the docking progress. Minimization was performed on 1,000 steps of Steepest Descent and then minimized by Conjugate Gradient. Different generated ligands poses were docked into the defined binding site on the RbAp48 protein structure; ligand binding in the receptor cavity was evaluated by various scoring functions, including -PMF, -PMF04, and Dock Score.

### 2.3. Molecular Dynamics Simulation

The protein-ligand complexes were regarded as input structures in GROMACS 4.5.5 package [44] for molecular dynamic simulation; charmm27 force field was selected in the simulation system. The distance of the real space for box definition was set as 1.2 nm. The particle mesh Ewald (PME) method was used to treat Coulomb interactions as electrostatic. The Coulomb interaction between two charge particles was as follows:

(1) $$\begin{array}{c} V_{c}\left(r_{ij}\right)=f\frac{q_{i}q_{j}}{\varepsilon_{r}r_{ij}}. \end{array}$$

The cut-off distance of van der Waals (VDW) residues was set at 1.4 nm, using the following equation:

(2) $$\begin{array}{c} U\left(r\right)=4\varepsilon \left[{\left(\frac{\delta }{\gamma }\right)}^{12}-{\left(\frac{\delta }{\gamma }\right)}^{6}\right]. \end{array}$$

The linear constraint solver (LINCS) algorithm was used for fixing all bond lengths. The solvent setting for water simulation was based on the TIP3P model. Topology files and parameters of small compounds for docked ligands were generated by SwissParam web server [45]. We added Na and Cl ions to create a neutral system; the concentration of NaCl model was set to 0.145 M. 5,000 cycle steps of the steepest descent algorithm were used for energy minimization then followed by equilibration performed under position restraints for 1 ns under constant temperature dynamics (NVT type) conditions at a temperature of 310 K. Following this step, all production dynamics simulations were performed for 5,000 ps under constant pressure and temperature dynamics (NPT type). The temperature of the simulation system was set as 310 K. MD conformations were saved every 20 ps for trajectory, migration, and residues fluctuation analysis.

### 2.4. Molecular Dynamics Analysis

All MD conformations were analyzed under GROMACS 4.5.5 software, root mean square deviation (RMSD), and radius of gyration (Rg) by the commands g_cluster and g_gyrate, respectively. Total energy was calculated by the g_energy program. Root mean squared fluctuation (RMSF) of protein residues was obtained by g_rmsf. Mean square displacement (MSD) was performed using g_msd; the docked ligand was used to observe the migration over the simulation time. Cluster analysis was performed to the cluster docked ligand complex by g_cluster program. The method for cluster determination was the linkage algorithm.

## 3. Results and Discussion

### 3.1. Docking Results

**Figure 1:**
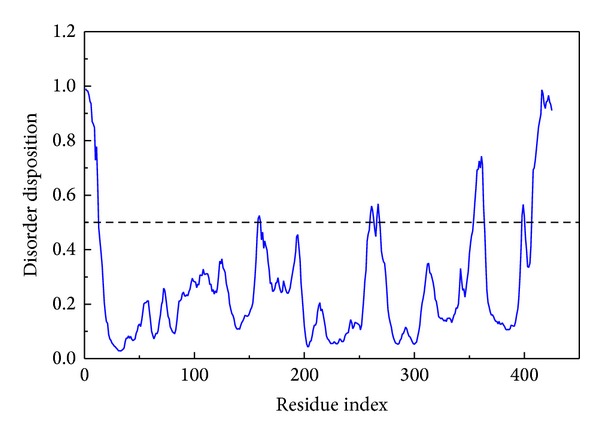
Disorder analysis of sequence of RbAp48 from the result of PONDR-FIT prediction. A value of disorder disposition above 0.5 in disorder disposition indicates disorder residues.

**Figure 2:**
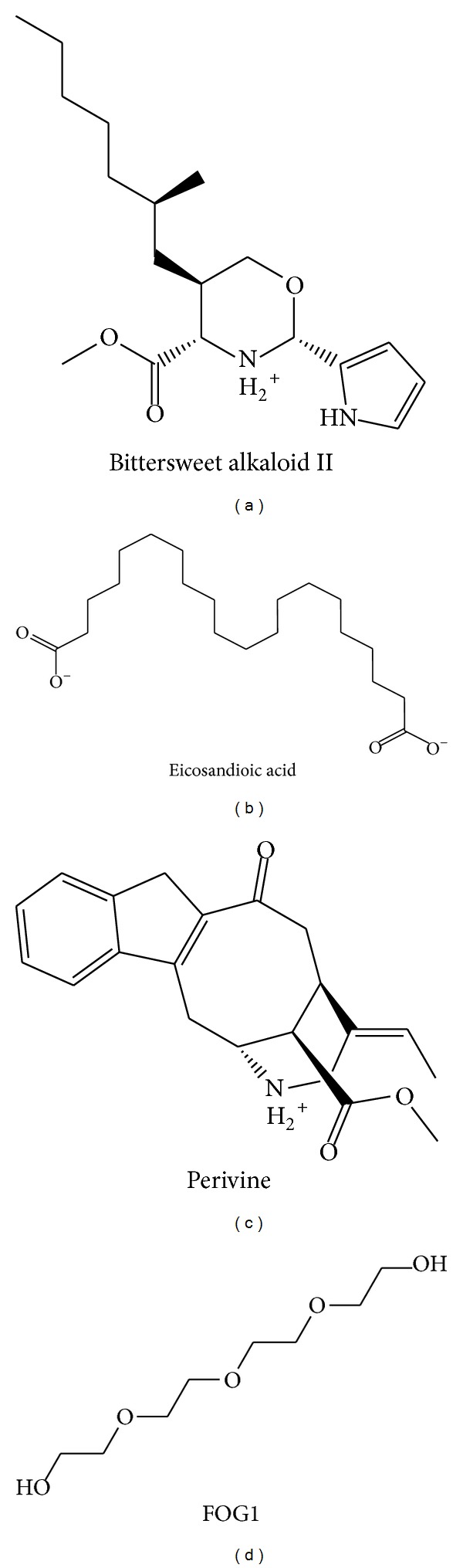
Chemical scaffolds of TCM candidates and control.

**Figure 3:**
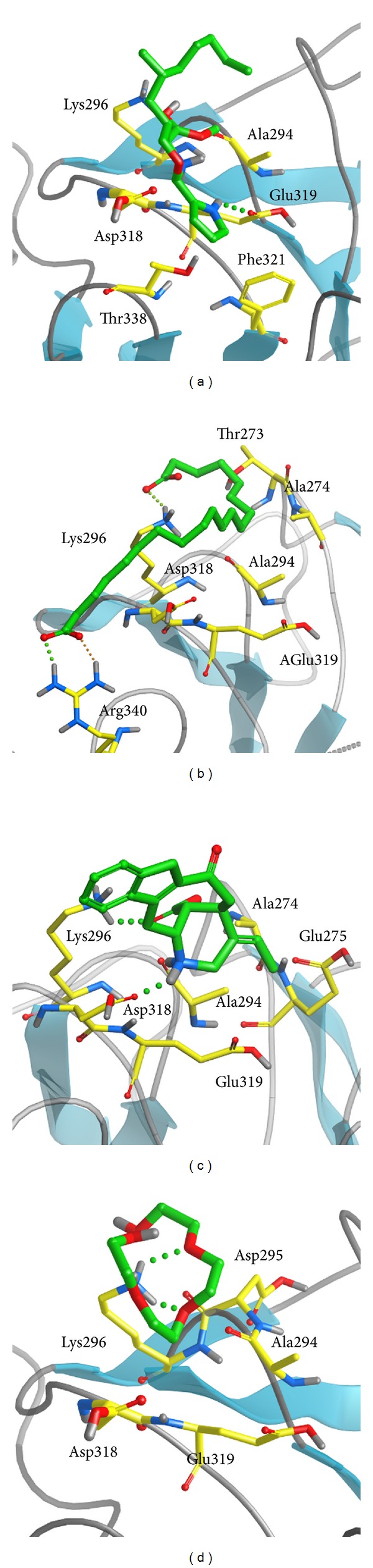
The docking poses of small compounds: (a) Bittersweet alkaloid II, (b) Eicosandioic acid, (c) Perivine, and (d) FOG1 in binding site of RbAp48. The small molecular and amino acids are colored in green and yellow, respectively.

We employed PONDR-FIT [43] to predict the order/disorder from the protein sequence, in order to understand if the binding region of RbAp48 is an order-folded structure. The sequence number of the major binding region was from 250 to 350 (Figure 1), and the values for disorder disposition were below 0.5, which indicated that the binding site of RbAp48 is a folded structure, and the ligand binding may not be affected by protein structure [46]. Docking analysis was based on -PMF, -PMF04, and Dock Score to evaluate the docking pose of traditional Chinese medicine (TCM) compounds. From the scoring analysis, FOG1 was regarded as a control for comparison; candidates with higher values of scores than FOG1 are shown in Table 1. For ADMET evaluation, all the TCM candidates and FOG1 had no CYP2D6 inhibition; suggesting that CYP2D6 may not be affected by these ligands in the liver. The top TCM candidates displayed good absorption (absorption = 0), high or medium blood brain barrier (BBB) penetration (penetration = 1 or 2), and good drug-like solubility (−4.0 < solubility value < −2.0). FOG1 had moderate absorption (absorption = 1), undefined BBB penetration (penetration = 4), and low drug-likeness absorption (−6.0 < solubility value < −4.0). These data show that the top TCM candidates are more drug-like than the control. All docked ligands were ranked by Dock Score, and it was found that the Dock Score of the top TCM candidates (including score values of -PMF and -PMF04) was greater than FOG1. Furthermore, due to the score value of -PMF04 varying significantly between Perivine and Docosandioic acid, we selected Bittersweet alkaloid II, Eicosandioic acid, and Perivine for further study. The chemical scaffolds of the TCM candidates and the control are shown in Figure 2. Docking poses of Bittersweet alkaloid II displayed H-bond with Glu319; close residues include Lys296, Asp318, Thr388, Phe321, and Ala294 (Figure 3(a)). For Eicosandioic acid, there are two amino acids (Arg340 and Asp295) which form H-bond interactions with the ligand; the surrounding residues are Asp318, Glu319, Ala294, Ala274, and Thr273 (Figure 3(b)). Perivine has two amino acids that generate H-bonds for ligand binding: Lys296 and Asp318; the amino acids Glu319, Glu275, Ala294, and Ala274 are near the docked ligand (Figure 3(c)). For the docked pose of FOG1, only Lys296 of RbAp48 can generate H-bond interactions; close residues include Asp318, Glu319, Ala294, and Asp295 (Figure 3(d)). It is worth noting that Ala294 is the common residue for each ligand binding, and the result reveals that all the small compounds were bound in the same region of RbAp48. In a further study, molecular dynamics simulation was utilized to analyze the variation of each ligand in the protein structures.

### 3.2. Stability Analysis of the Dynamics Complexes

**Figure 4:**
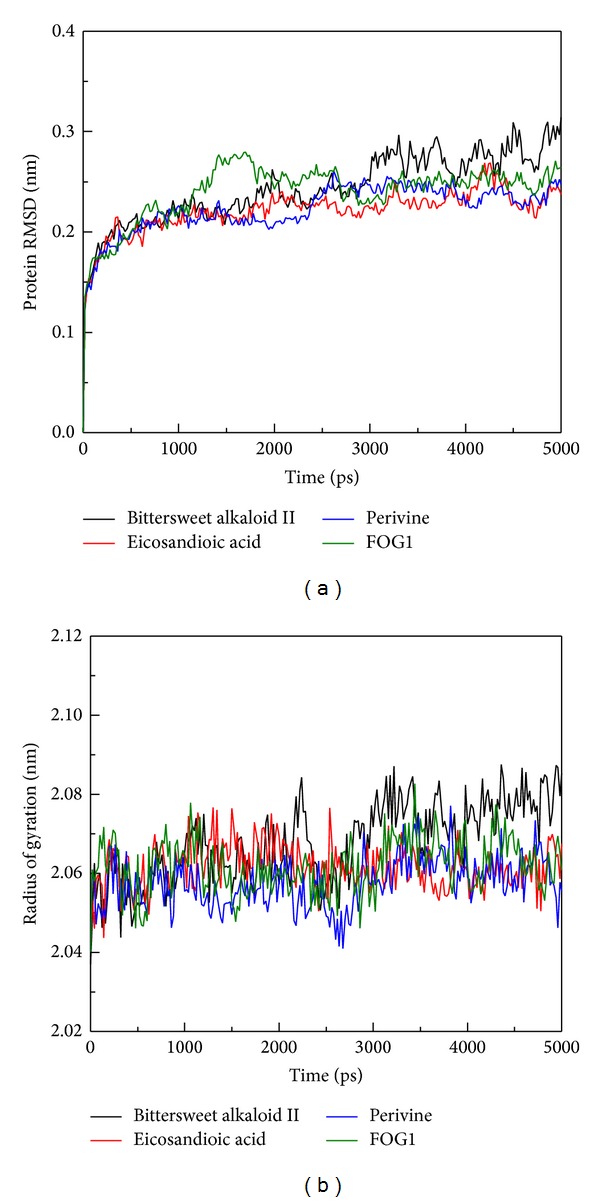
Plots of (a) Protein RMSD and (b) radius of gyration from protein-ligand complexes for a simulation time of 5,000 ps.

The RMSD value of protein atoms and the Rg value were used to analyze the stability of the protein structure. The value of protein RMSD was between 0.2 and 0.3 nm from 1,000 to 5,000 ps (Figure 4(a)); substantial fluctuations were not observed indicating that all conformations are stable after a simulation time of 1,000 ps. For Rg's plot evaluation, the complex with Bittersweet alkaloid II is slightly increased from 2,000 to 5,000 ps, but the Rg value does not move away from the initial value (Figure 4(b)). The value for the Rg complex with Eicosandioic acid, Perivine, and FOG1 remained constant during a simulation time of 5,000 ps, which revealed that the protein structure is compact after MD simulation. Bittersweet alkaloid II may affect the structure of RbAp48, but Rg's plot shows that the complex remained stable from 3,000 to 5,000 ps.

**Figure 5:**
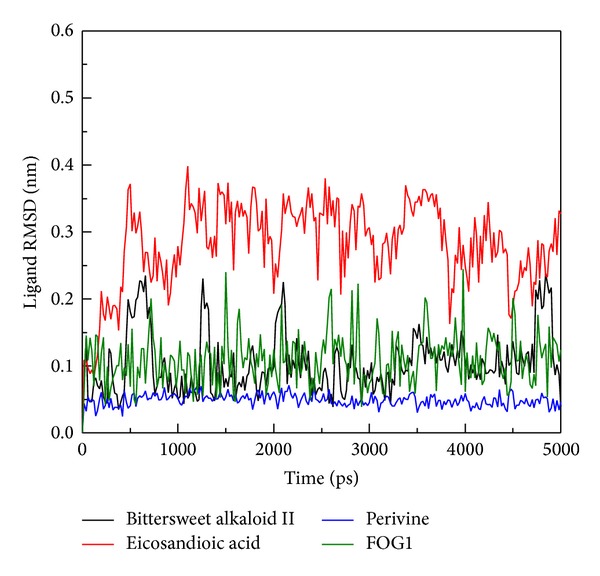
Plot of Ligand RMSD values from protein-ligand complexes for a simulation time of 5,000 ps.

**Figure 6:**
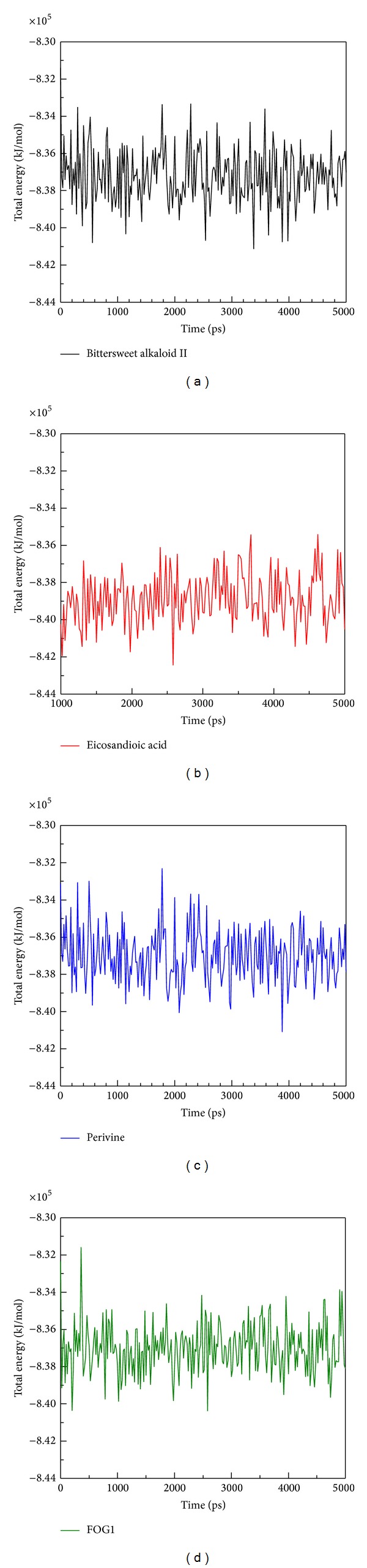
Total energy of RbAp48 with docked ligand: (a) Bittersweet alkaloid II, (b) Eicosandioic acid, (c) Perivine, and (d) FOG1 for a simulation time of 5,000 ps.

The RMSD of each small molecule during MD simulation (Figure 5) was also analyzed, and the Ligand RMSD of Eicosandioic acid was found to show large fluctuations at 500 ps and tended to an average of 0.25 nm from 500 ps to the end of the simulation. Bittersweet alkaloid II had similar fluctuations to FOG1, with an average of 0.1. The most stable ligand RMSD was found in Perivine, which was constant among all MD simulations. No significantly increased values were observed for total energy analysis in any complexes during a simulation time of 5,000 ps (Figure 6). The total energy remained around −8.35 × 10^5^ kJ/mol for Bittersweet alkaloid II, Perivine, and FOG1. In contrast, Eicosandioic acid had the lowest total energy, the average being −8.39 × 10^5^ kJ/mol. These results suggest that all structures of the complexes remained constant during MD simulation, and the binding regions do not display structural flexibility.

### 3.3. Stability Analysis of Residues on the Major Binding Region during MD Simulation

**Figure 7:**
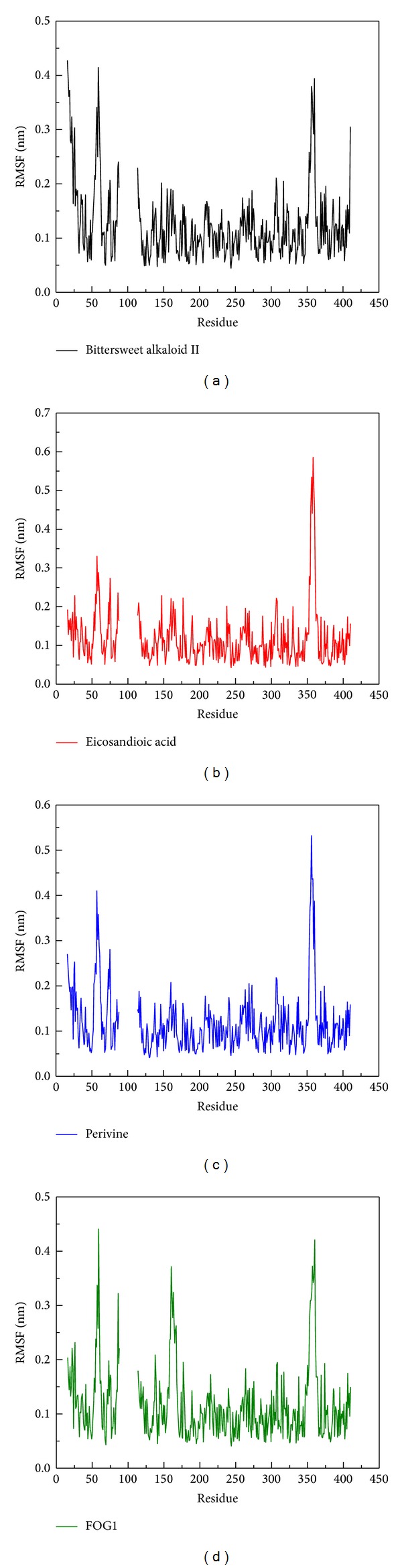
RMSF values of each residue of RbAp48 with docked ligand: (a) Bittersweet alkaloid II, (b) Eicosandioic acid, (c) Perivine, and (d) FOG1 ligand for a simulation time of 5,000 ps.

**Figure 8:**
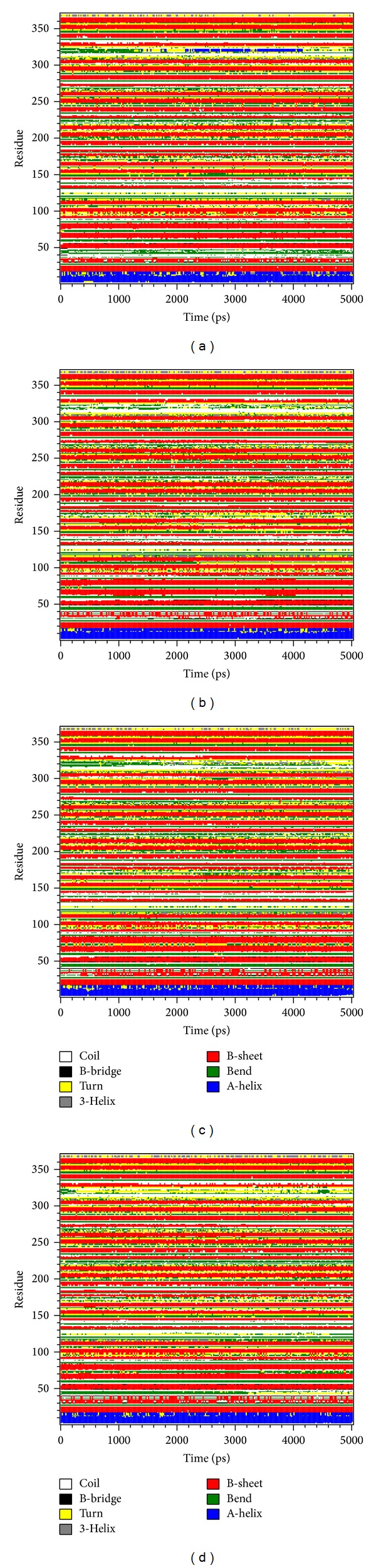
DSSP analysis of complexes with ligands: (a) Bittersweet alkaloid II, (b) Eicosandioic acid, (c) Perivine, and (d) FOG1.

**Figure 9:**
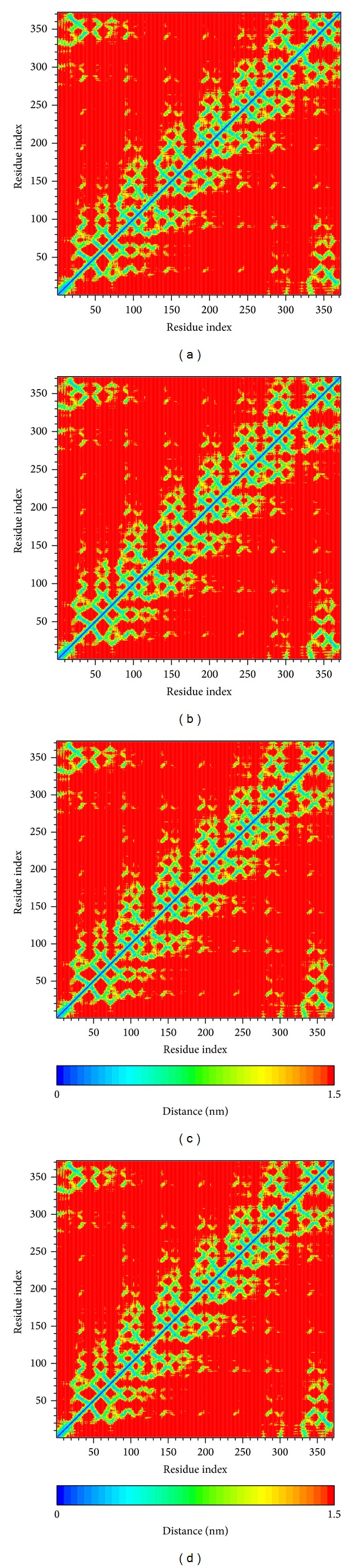
Matrix of smallest distance between each pair of amino acids in complex with (a) Bittersweet alkaloid II, (b) Eicosandioic acid, (c) Perivine, and (d) FOG1.

We calculated the RMSF of each residue to analyze the flexibility of residues on protein structure. The major binding region (from 250 to 350 residues) showed no significant increment in structure of RbAp48 with all ligands (Figure 7). From DSSP analysis, all complexes remained exist helices and beta-sheets during a simulation time of 5,000 ps (Figure 8). We also calculated the distance per pair of each residue for 5,000 ps. The matrix of smallest distance between each pair of amino acids showed that there were no distinct changes for all protein-ligand complexes (Figure 9). The results reveal that the structure of RbAp48 remained stable during all MD simulations.

### 3.4. Migration Analysis of Ligands in Protein Binding Site

**Figure 10:**
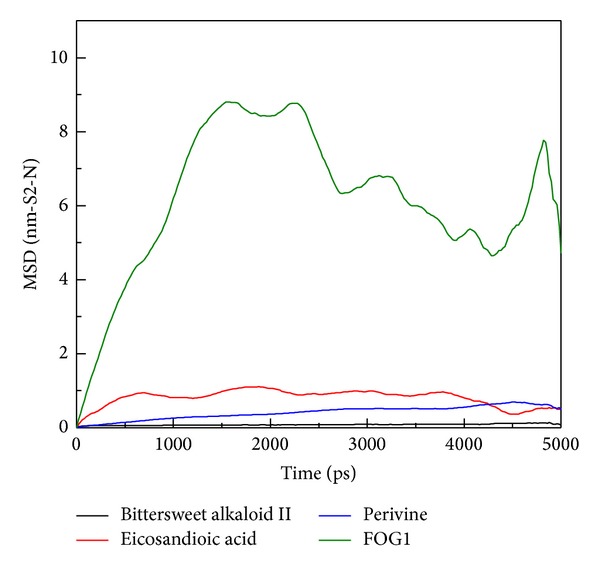
Mean square displacement (MSD) of different ligands for a simulation time of 5,000 ps. The value of MSD indicates migration of ligand from the initial site.

**Figure 11:**
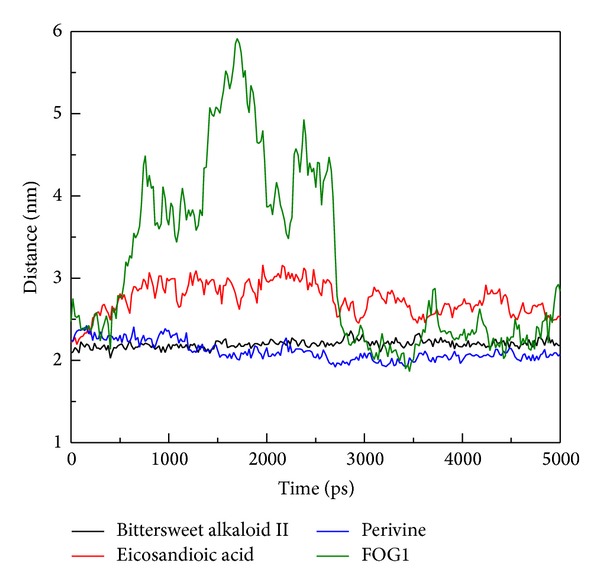
Distance between centers of mass of RbAp48 and each ligand for a simulation time of 5,000 ps.

MSD was used to measure the migration of the docked ligand during MD simulation in order to assess the variation of each ligand after docking into the protein binding site. The MSD value of FOG1 was the most distinctive and displayed a rapid increase during initial simulation to the end of the 5,000 ps (Figure 10). All TCM candidates still had an MDS value below 1 nm. These results suggest that the docking poses were not changing the binding position significantly during the simulation time. In further study, the distances between the center mass of the protein and each ligand were measured for all simulation times to understand the movement of the docked compounds. Interestingly, FOG1 was found to have a large protein-to-ligand distance of 500 to 3,000 ps (Figure 11), indicating that FOG1 was moving away from the initial binding position and transferring to another site in the protein structure. For the three TCM compounds, there were no substantial fluctuations in movement, suggesting that each ligand could bind stably in the RbAp48 structure.

### 3.5. Snapshots Analysis and Ligand Channel Prediction

**Figure 12:**
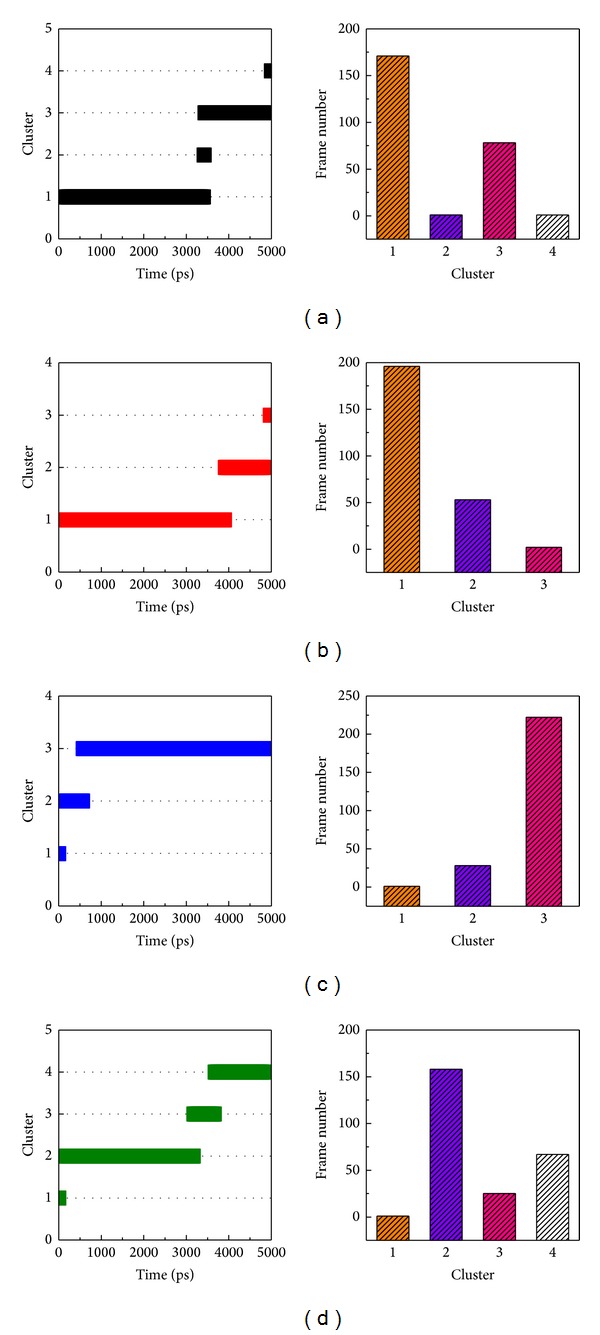
Clustering analyses of all conformations for a simulation time of 5,000 ps.

**Figure 13:**
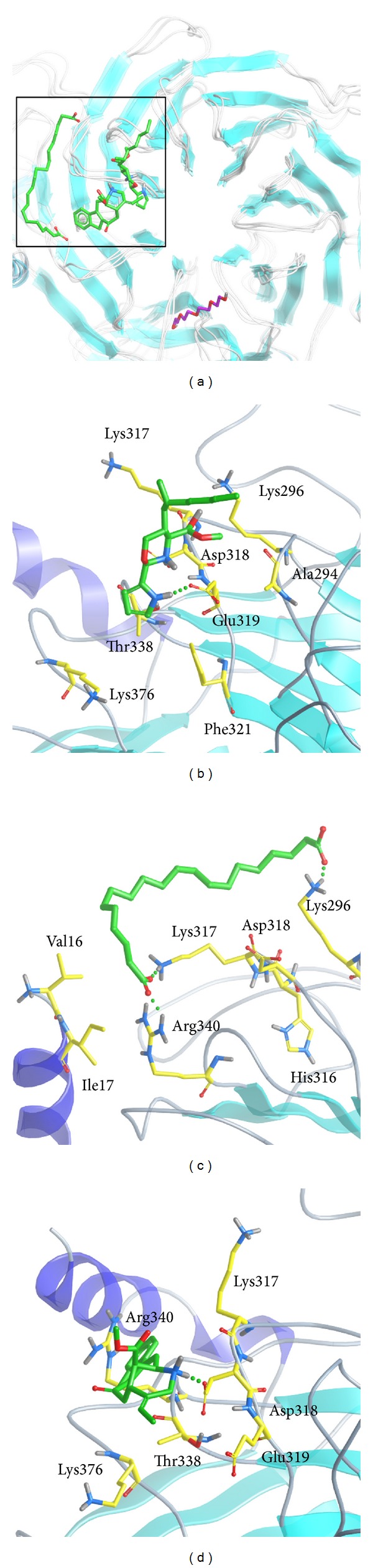
(a) Docking position of TCM candidates in superimposition of each representative conformation from MD simulation; the TCM compounds are colored in green. The docking poses of each TCM compound: (b) Bittersweet alkaloid II, (c) Eicosandioic acid, and (d) Perivine in RbAP48 binding site.

**Figure 14:**
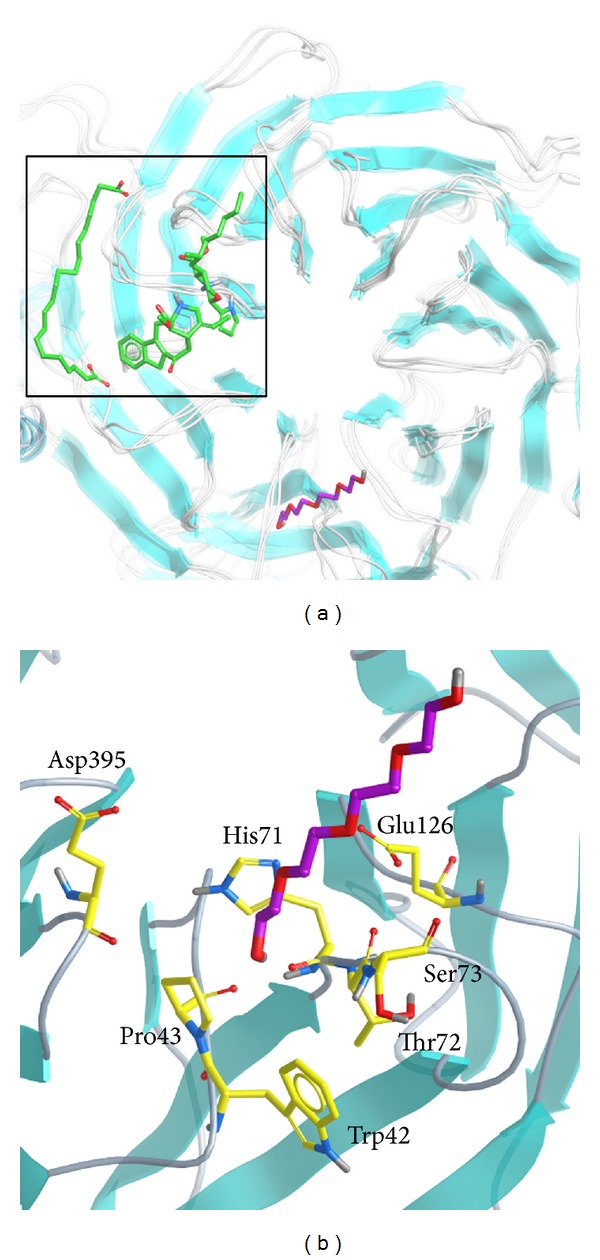
Docking position (a) and binding pose (b) of FOG1 in superimposition conformation. FOG1 is colored purple.

**Figure 15:**
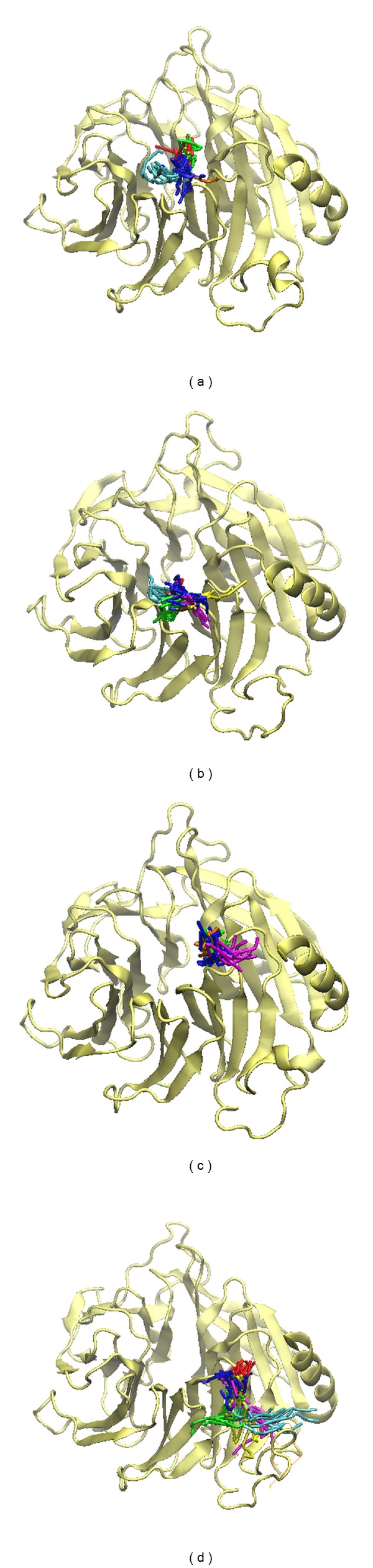
Ligand channel prediction of docked ligand in RbAp48: (a) Bittersweet alkaloid II (b) Eicosandioic acid, (c) Perivine, and (d) FOG1.

In order to identify the most stable structure during the entire MD simulation and to understand the movement of FOG1, all conformations from MD simulation were clustered into three or four groups (Figure 12). The middle conformation from the final groups of clusters was chosen, and each middle frame is listed in Table 2. The protein structures were then superimposed on each middle frame (Figure 13(a)). The position of FOG1 was found to be far from other three candidates due to the other three candidates not migrating significantly, but remaining close to the initial docking positions. The three candidates have common residues for ligand binding, Bittersweet alkaloid II generated one H-bond with E319 (Figure 13(b)), Eicosandioic acid had two H-bonds interacting with Arg340 and Lys276 (Figure 13(c)), and Perivine had one H-bond with Asp318 (Figure 13(d)). We found that K317, D318, and E319 can form H-bonds with the TCM candidates. In the initial docking poses, K317, D318, and E319 interacted with TCM compounds and FOG1, which illustrates that the docked ligands are not variable after MD simulation. From the FOG1 snapshot analysis, it can be seen that the docked ligand migrates significantly from the initial pose to the other site on RbAp48 (Figure 14(a)). Lys296 forms H-bonds with FOG1 in the initial docking pose, but the surrounding residues changed to Glu395, His71, Pro43, Trp42, Glu126, Ser73, and Thr72 in the representative snapshot (Figure 14(b)). These results show that FOG1 is relatively more flexible than the other TCM compounds. In addition, we also predicted migration channel of each docked ligand during simulation time of 5000 ps; the prediction results were shown in Figure 15. The prediction of FOG1 displayed long distance channel than other three TCM compounds; the funding is correlated with snapshots analysis and migration analysis and illustrated that the TCM candidates could form stable binding conformation to interact with RbAp48.

## 4. Conclusion

From ADMET and docking analysis, our candidates are determined to be more drugs-like than FOG1, and the three scoring functions -PMF, -PMF04, and Dock Score are higher than the control. In migration analysis after MD simulation, FOG1 displayed low stability for RbAp48 binding, which was correlated with the low affinity in the docking results. The structure of RbAp48 did not change significantly during MD simulation, suggesting that FOG1 migration was not effected by protein structure. The unstable RbAp48-FOG1 complex could reduce the transcription function. The top three candidates Bittersweet alkaloid II, Eicosandioic acid, and Perivine bound stably in the binding site of RbAp48 and did not change the binding positions from the initial docking poses. Our results indicate that these TCM compounds may have potential for the design of novel drugs to solve the unstable RbAp48-FOG1 complex problem and provide a new mechanism for AD therapy.

## Figures and Tables

**Table 1:** Top ten candidates and control.

Name	-PMF	-PMF04	Dock score	Absorption^a^	BBB level^b^	CYP2D6^c^	Solubility^d^
Bittersweet alkaloid II	**48.19**	**36.70**	**98.838**	**0**	**2**	**0**	**3**
Eicosandioic acid	**48.52**	**20.37**	**97.934**	**0**	**2**	**0**	**3**
Perivine	**30.39**	**23.90**	**95.328**	**0**	**2**	**0**	**3**
Docosandioic acid	34.37	17.84	95.194	0	1	0	3
N-Methylmescaline	33.25	30.41	93.733	0	2	0	4
Isolobelanine	38.74	22.15	93.190	0	1	0	3
5-Methoxy-N-methyltryptamine	24.72	22.30	89.676	0	2	0	4
Vincamine	31.63	28.02	89.338	0	2	0	3
Quininone	35.22	26.12	88.540	0	2	0	3
FOG1∗	**4.65**	**14.08**	**67.647**	**1**	**4**	**0**	**5**

*Control.

^a^Absorption: good absorption: 0; moderate absorption: 1; low absorption: 2.

^b^BBB level (blood brain barrier): high penetration: 1; medium penetration: 2; low penetration: 3; undefined penetration: 4.

^c^CYP2D6: noninhibitor < 0.5; inhibitor > 0.5.

^d^Solubility: −6.0 < value < −4.0 indicates low drug-likeness compounds; −4.0 < value < −2.0 indicates good drug-likeness compounds; −2.0 < value = 0.0 indicates optimal drug-likeness compounds; 0.0 < value indicates being too soluble and with no drug-likeness.

**Table 2:** Time of middle structure in each cluster from all MD conformations.

Cluster	Time of middle frame (ps)			
	Bittersweet alkaloid II	Eicosandioic acid	Perivine	FOG1
1	2320	1760	0	0
2	3420	4380	320	2000
3	3820	4980	2300	3400
4	5000	—	—	4480
